# Early, Non-Invasive Sensing of Sustained Hyperglycemia in Mice Using Millimeter-Wave Spectroscopy

**DOI:** 10.3390/s19153347

**Published:** 2019-07-30

**Authors:** Aldo Moreno-Oyervides, Pedro Martín-Mateos, M. Carmen Aguilera-Morillo, Giacomo Ulisse, María C. Arriba, María Durban, Marcela Del Rio, Fernando Larcher, Viktor Krozer, Pablo Acedo

**Affiliations:** 1 Department of Electronic Technology, Universidad Carlos III de Madrid, Leganes, 28911 Madrid, Spain; 2 Instituto de Investigaciones sanitarias de la Fundación Jiménez Díaz (IIS-FJD), 28040 Madrid, Spain; 3 Department of Statistics, Universidad Carlos III de Madrid, Leganes, 28911 Madrid, Spain; 4 Santander Big Data Institute, Universidad Carlos III de Madrid, Getafe, 28903 Madrid, Spain; 5 Physics Institute, Goethe University Frankfurt am Main, 60438 Frankfurt am Main, Germany; 6 Centro de Investigación Biomédica en Red de Enfermedades Raras (CIBERER), 28029 Madrid, Spain; 7 Epithelial Biomedicine Division, CIEMAT, Avenida Complutense 40, 28040 Madrid, Spain; 8 Department of Bioengineering, Universidad Carlos III de Madrid, Leganes, 28911 Madrid, Spain

**Keywords:** millimeter-wave spectroscopy, sustained hyperglycemia, non-invasive diagnosis techniques, early diabetes detection, functional principal component analysis

## Abstract

Diabetes is a very complex condition affecting millions of people around the world. Its occurrence, always accompanied by sustained hyperglycemia, leads to many medical complications that can be greatly mitigated when the disease is treated in its earliest stage. In this paper, a novel sensing approach for the early non-invasive detection and monitoring of sustained hyperglycemia is presented. The sensing principle is based on millimeter-wave transmission spectroscopy through the skin and subsequent statistical analysis of the amplitude data. A classifier based on functional principal components for sustained hyperglycemia prediction was validated on a sample of twelve mice, correctly classifying the condition in diabetic mice. Using the same classifier, sixteen mice with drug-induced diabetes were studied for two weeks. The proposed sensing approach was capable of assessing the glycemic states at different stages of induced diabetes, providing a clear transition from normoglycemia to hyperglycemia typically associated with diabetes. This is believed to be the first presentation of such evolution studies using non-invasive sensing. The results obtained indicate that gradual glycemic changes associated with diabetes can be accurately detected by non-invasively sensing the metabolism using a millimeter-wave spectral sensor, with an observed temporal resolution of around four days. This unprecedented detection speed and its non-invasive character could open new opportunities for the continuous control and monitoring of diabetics and the evaluation of response to treatments (including new therapies), enabling a much more appropriate control of the condition.

## 1. Introduction

Over the years, technological advances have led to the enhancement of medical diagnosis, treatments and procedures, and improving quality of life for patients [1,2,3]. In this regard, several efforts have focused on diabetes mellitus (DM) sensing and monitoring [4]. DM is a very challenging disease affecting an increasing part of the world population every year; it is estimated that there will be 366 million cases in 2030 [5,6]. Diabetes encompasses a set of metabolic conditions characterized by the presence of high blood glucose levels (BGL), i.e., chronic or sustained hyperglycemia [7]. It is well known that persistent exposure to hyperglycemia on the body can lead to cardiovascular diseases and damage to the kidneys, nerves, and eyes, among other organs [8,9,10,11]. Therefore, diabetes sensing and detection in its earliest stages are critical to combating the disease reducing serious health complications.

Nowadays, the diagnosis and follow-up of diabetes are based on classical enzymatic methods, such as plasma glucose (e.g., Fasting Plasma Glucose (FPG) or Oral Glucose Tolerance Test (OGTT)), and glycohemoglobin (A1c or HbA1c) measurements [12,13,14]. All these methods are conducted under well-stablished protocols and standards for diagnostic criteria [15]. It is generally considered that HbA1c gives an indication of the average blood glucose content during the last three months, being less influenced by abrupt changes in glycemia. Unfortunately, both plasma glucose and HbA1c tests are invasive sensing techniques (blood samples are required) and HbA1c tests also depend on complex equipment only available at specialized laboratories, increasing the number of undiagnosed cases of diabetes and mortality rate due to lack of adequate treatment [16]. 

There are novel test methods for diabetes detection and monitoring focused on non-invasive blood glucose measurement based on IR technologies [17,18,19], which can be linked to interstitial fluid detection using spectroscopic sensing. These methods suffer from the cross-influence of the skin properties (skin moisture etc.) and predisposition of the tissue. Other approaches for non-invasive blood glucose level sensing, such as reverse iontophoresis, fluorescence, bioimpedance, photoacoustic, and ocular spectroscopy, etc. have also been explored, but exhibit disadvantages such as poor accuracy, skin irritation, variation of glucose readings in different individuals, high sensitivity to environmental parameters, difficulty in calibration, and high costs [20,21].

In this paper, which continues our previous work for the non-invasive detection of sustained hyperglycemia in animal models [22], a different approach is proposed for diabetes detection and monitoring based on the non-invasive sensing of metabolic states and detection of gradual changes of metabolism during sustained hyperglycemia. 

The motivation to employ millimeter-wave radiation for biomedical applications originates from its capability to penetrate and sense without harm the biological tissue and structures. In particular, the millimeter-wave frequency range exhibits great potential for the non-invasive blood glucose level monitoring [23,24,25,26] and non-invasive, in-vivo blood glucose monitoring in animal models, which has been demonstrated at frequencies below 40 GHz [27]. When considering penetration through the skin, even higher frequencies can be considered, as verified by the authors [22,28]. The advantage of using higher frequencies is that the interaction region is well defined, the probing location is smaller due to tighter focusing capabilities or smaller waveguide dimensions, dispersion effects are less pronounced, and compactness of the sensing equipment is improved. However, excessively high frequencies beyond millimeter-waves exhibit poor signal-to-noise ratio and, therefore, long measurement times, which are not compatible with in vivo monitoring. Therefore, the authors have chosen the W-band (75–110 GHz) as a compromise between penetration depth, interaction volume, and signal to noise ratio. 

In our previous work, the hyperglycemia condition was clearly discriminated from normoglycemia and two different types of hyperglycemia were detected: Hyperglycemia caused by overfed animals (obese mice) and hyperglycemia due to diabetes condition (diabetic mice). In addition, it was proven that such discrimination using millimeter-wave spectroscopy sensing was not influenced by the instantaneous glucose level, skin thickness, or skin phenotypes [22,28]. The results indicate that the proposed method is a very powerful tool for sustained hyperglycemia and diabetes detection being both non-invasive and highly independent of environmental conditions. The millimeter-wave (mm-wave) spectroscopy system is a highly reliable and stable instrument that can be realized as very compact and cost-effective. The actual measurement time is in the order of seconds and is determined by the spectrum acquisition time. The data processing could in principle operate in real time. 

The biological samples studied here involve numerous metabolites and substances, which resonate at different partially overlapping frequencies when probed with electromagnetic waves. Therefore, the measured spectral response is analyzed here using a non-targeted metabolomic approach [29,30,31]. The non-targeted metabolomics approach focuses on the association of a target pathology with a characteristic spectral response in a certain frequency band without the necessity to quantify an isolated metabolite.

In this work, we provide evidence that the same approach can be employed with high accuracy for the early detection and monitoring of the hyperglycemia typically associated with diabetes, with unparalleled speed and time resolution. We refer here to these studies as longitudinal studies of hyperglycemia. The development of hyperglycemia in mice with induced diabetes could be identified even before the full diabetic state has been established. These results show that the sensing principle is very sensitive to metabolic changes associated to sustained hyperglycemia and this, consequently, demonstrates its great potential for both the early non-invasive detection of diabetes and the accurate monitoring of the disease. In fact, it enables the following of individuals in a timeline across the dynamic evolution of the diabetic state. In this paper, a preliminary validation test has been performed using a classifier based on functional principal components for hyperglycemia typically associated with diabetes. A validation test had been carried out on a population of 12 mice with six diabetic mice and six control mice (normoglycemic mice) obtaining a correct classification rate of 100%. Then, 16 mice with drug-induced diabetes were studied at different moments of their condition using the same predictor. These results have been evaluated to identify the mechanisms behind the metabolic sensing capabilities for monitoring the early development of hyperglycemia along 14 days after completion of the diabetes induction process.

## 2. Materials and Methods

### 2.1. Sample Population

All the measurements were carried out on a group of mice representative of different glycemic states. Sample population was designed considering two important targets: Validate discrimination of hyperglycemia, typically associated with diabetes, on a test sample by using a classifier based on functional principal components, and, more importantly, study the spectral response over time on transition cases from normoglycemia to hyperglycemia, which determines the time resolution of the sensing approach.

There are 2 main classes in the sample: Normoglycemia and hyperglycemia. Healthy mice were considered as normoglycemic cases and diabetic mice genetically mutated to be insulin-resistant Lep^db^/Lep^db^ [32] as hyperglycemic cases. In addition, mice with drug-induced diabetes (diabetized) were incorporated in the study, in order to evaluate the sensing performance of drug-induced diabetes. The diabetes induction process involved mice that received 3 intraperitoneal injections of Streptozotocin (Sigma-Aldrich, Inc., St. Louis, MO, USA) with concentrations of 0.1 mg/g, 0.1 mg/g, and 0.15 mg/g during a period of 5 days.

For the validation test, 2 samples from the mice population (training sample and test sample) were employed and measured separately. Two experiments were performed at different times. The training sample corresponded to the sample of the mice population measured and presented in our last publication [22]. The test sample was measured in a second experiment presented in the current work. Both the training sample and test sample are detailed in Table 1.

In the second experiment, 16 diabetized mice were also measured simultaneously with the 6 diabetic mice and 6 healthy mice (test sample) as reference. The experiment lasted 19 days and 4 measurements were performed. The measurements started on the last day of the treatment (animals receiving the final dose) and finished 14 days hereafter. During the experiment, 6 diabetized mice died before the 3rd measurement (10 days after treatment) due to the adverse effects of the drug [33].

All animals were purchased from Elevage-Janvier (Le Genest-Saint-Isle, France) and housed individually in pathogen-free conditions at the Centro de Investigaciones Energéticas, Medioambientales, y Tecnológicas (CIEMAT) Laboratory Animals Facility (Spanish registration number 28079-21 A).

### 2.2. Experimental Protocol

All experimental procedures were carried out according to European and Spanish laws and regulations (European convention ETS 1 2 3, about the use and protection of vertebrate mammals used in experimentation and other scientific purposes, Directive 2010/63/UE and Spanish Law 6/2013, and R.D. 53/2013 about the protection and use of animals in scientific research). Procedures were approved by the Animal Experimentation Ethical Committee of the CIEMAT according to all external and internal bio-safety and bio-ethics guidelines, and by Spanish competent authority with registered number PROEX 176/15.

**Figure 1:**
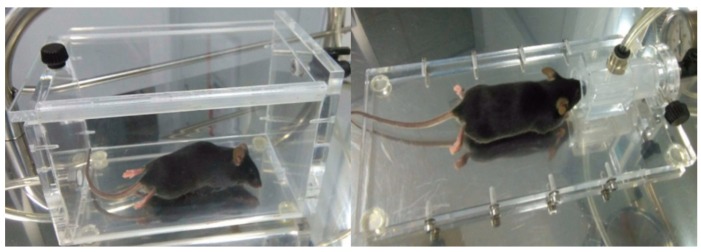
Pictures of the anesthetic induction. The mouse was anesthetized into an induction chamber (left panel). Then, the animal was placed on a tray with its snout into a supplying line of anesthesia (right panel) until the measuring process ended.

Firstly, it is important to highlight that the body hair from the back of the mice was regularly cut off to facilitate the handling of their skin. Besides this treatment, no special procedures were installed for the measurements. It has been proven earlier that the sensing approach was insensitive to the skin properties of the animals such as hairs [22,28]. Excessive movement of the mice was prevented using anesthesia during the measurement time (around 45 s). Anesthesia was administered by inhalation (isoflurane); each mouse was introduced into an induction chamber with isoflurane mixed with oxygen (3–4% of isoflurane concentration) until anesthetic takes effect, then, it was placed on a table to continuously inhale a lower anesthesia concentration during the whole measurement (3–13.5% of isoflurane concentration) via a mask (see Figure 1). The use of isoflurane has the advantages of lower time for induction and awakening and was less harmful for mice.

**Figure 2:**
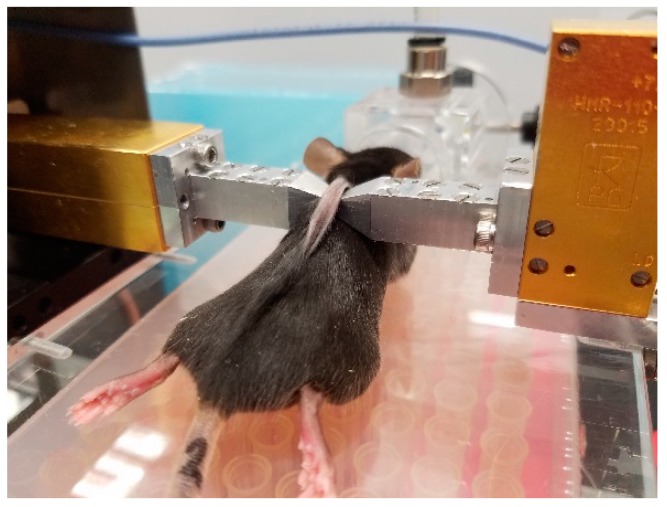
Photograph taken during the spectroscopic measurement.

The spectroscopic measurement was taken directly on a skin fold of the mice (non-invasively). A skin fold on the mice back was placed between 2 previously aligned straight cuts of a WR10 waveguide, as shown in Figure 2. In this way, we have ensured the propagation of the signal through the skin and underlying layers, resulting in an interaction between the emitted waves and the skin fold during the spectroscopic measurement. The mm-wave spectrum was acquired in the frequency range between 75 GHz to 111 GHz in steps of 1.5 GHz as described below. The setup was capable of amplitude and phase measurements of the reflection and transmission coefficients, respectively. We have used the amplitude of the transmission coefficient only because the transmitted wave was less sensitive to the outer skin layers and propagated through all the biological tissue. We have proven earlier [28] that the amplitude and phase were important if one did not employ statistical techniques. The major advantage of the Principal Component Analysis (PCA) technique employed here was that the sensing involves only amplitude data. The distance between the straight cuts was carefully adjusted to fix the skin without infringing damage or pain to the mice (800 µm). However, it should be noted that the pressure of the 2 waveguide straights on the mice skin was not of prime importance in the diagnostic procedure. Prior to the experiment, several tests were carried out to avoid receivers’ saturation by adjusting input signal power levels at each measured frequency.

### 2.3. Non-Invasive mm-Wave Spectroscopy System for Assessment of Glycemic States

**Figure 3:**
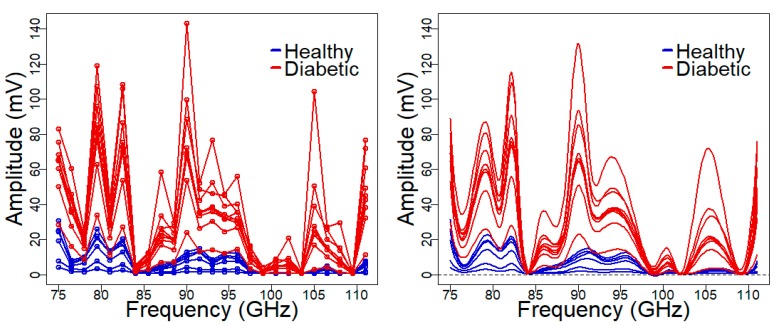
Measured amplitude transmittance spectrum. Raw spectral data collected by the spectroscopy system (**left panel**), and the estimated curves using regression splines with a cubic B-spline basis defined on 17 breakpoints (**right panel**).

All measurements were carried out by using a spectroscopy system [22], which is briefly described here. A frequency sweep is generated in the Ku band (12.5–18.5 GHz in steps of 250 MHz) by using an APSYN420 (AnaPico, Zurich, Switzerland) synthesizer, then, the frequency was multiplied by a factor of 6 to reach the W-band frequencies (75–111 GHz) by an AFM6-110 Active Frequency Multiplier (Radiometer Physics GmbH, Meckenheim, Germany). Before reaching the skin fold, a directional coupler was introduced and the reflected and the reference signals were measured using harmonic receivers, respectively. The transmittance was measured after the sample by a direct connection of a harmonic receiver. All harmonic receivers were HMR-110-6 W-band (Radiometer Physics GmbH, Meckenheim, Germany). The output from each receiver was at an intermediate frequency of 3.6 MHz and was digitized with a bandwidth of 10 MHz by an acquisition card (Handyscope HS4-10, TiePie engineering, Sneek, Netherlands). The acquisition and processing of the data during the measurement process was performed using LabVIEW. The amplitude spectra of the transmitted waves acquired by the spectroscopic system at the first measurement day of the experiment are shown in Figure 3 (left panel).

### 2.4. Functional Data Analysis for Spectral Data

The raw sensing data were processed using functional data analysis (FDA), which is a current topic in statistical mathematics of continuous signals. The digital acquisition of data implies that the spectral data were observed at a discrete set of frequencies thus that the sample data were given by $\left\{x_{ij}\;:\;i=1,\;.\;.\;.\;,n;\;j\;=\;1,\;.\;.\;.\;,\;m\right\},$ where n is the number of observations and m the number of independent variables (measured frequencies). However, the underlying process was continuous in frequency, which indicated that the appropriate statistical techniques to analyze this kind of data was FDA. FDA allows one to analyze the spectra as a continuous response along the whole frequency measurement interval rather than a set of discrete and independent frequencies.

The first step in the FDA is to obtain a functional data set from the spectra measured at discrete frequency points (raw data, no calibration required). To this end, the spectra are assumed to belong to a finite-dimensional space generated by a basis $\left\{\varphi_{1}\left(f\right),\ldots ,\varphi_{p}\left(f\right)\right\}$, thus that the i-th curve is given by $x_{i}\left(f\right)=\sum_{j=1}^{p}a_{ij}\varphi_{j}\left(f\right),\;f\in F,i=1,\ldots ,n$, with F being the frequencies interval. Finally, the basis coefficients $a_{ij}$ are estimated by using least squares smoothing with cubic B-spline [34]. This approximation is known as regression splines [35]. The degree of smoothness in the functional data set can be easily controlled by the dimension of B-spline basis, with smoother curves for lower dimensions. The raw data and the estimated regression splines (using 17 internal breakpoints to define the B-spline basis) are shown in Figure 3 (left and right panel, respectively).

The aim of this analysis was the discrimination of spectra according to their glycemic states (normoglycemia and hyperglycemia). Taking into account the small sample size, regression models are not feasible. As an alternative, we proposed a classifier based on Functional Principal Component Analysis (FPCA) [36].

FPCA is an extension of the classical PCA (for multivariate data) to the FDA context. The functional principal components are generalized linear combinations of the curves, uncorrelated and with maximum variance. In general, the j-th principal component scores are given by $\xi_{ij}=\int_{F}x_{i}\left(f\right)w_{j}\left(f\right)df,i=1,\;.\;.\;.,\;n,$ where $f_{j}$ is the weight function or loading, obtained such as to maximize the variance $var\left(\xi_{j}\right)=var\;[\int_{F}x_{i}\left(f\right)w\left(f\right)df]$ under the following constraints ${\left\|w\right\|}^{2}=1$ and $\int w_{l}\left(f\right)w\left(f\right)df=0,$ l=1,…,j−1. In addition, FPCA permits detection of a minimum interval of frequencies achieving the highest classification score. Another contribution of FPCA, with respect to classical PCA, is the application of the Karhunen-Loève decomposition, leading to a reduced set of q functional principal components, such as $x_{i}^{q}\left(f\right)=\sum_{i=1}^{q}\xi_{ij}w_{j}\left(f\right)$ whose explained variance is given by $\sum_{i=1}^{q}\lambda_{j}$, with $\lambda_{j}=var\left(\xi_{j}\right)$ and weight functions w(f). The principal component decomposition provides the best linear approximation of the sample curves in the least squares sense and should be used to simulate new spectra by reproducing the variability of the initial data set [37]. All the statistical analysis has been performed using the free statistical software R [38].

## 3. Results

As mentioned above, this work focuses on sensing the metabolic state during hyperglycemia in mice. Even more importantly, the work determines, how sensitive the proposed method is to detect glycemic changes associated with diabetes and analyzing the spectral response in the W-band. Hyperglycemia was first validated on an animal sample using functional principal component analysis. Subsequently, a group of mice with drug-induced diabetes was studied at different instances using the same methodology. The results obtained in both tests are shown below.

### 3.1. Blind (Predictive) Determination of Sustained Hyperglycemia in Animal Models

A sample of 20 mice of different strains representative of equally distributed normoglycemia and hyperglycemia cases have been defined as the training sample and the second sample of 12 mice has been taken as the test sample. Both samples have been described in more details in Section 2.1. A classifier based on functional principal components obtained from the training sample was used to predict on the test sample, as indicated in Section 2.4. The spectral measurements were performed in the W-band using transmit spectroscopy sensing instrument described in Section 2.3. The assessment of glycemic states was undertaken using the transmit amplitude through a folded skin on the back of the mice (see Section 2.2), referred to the reference channel amplitude. The spectra have been approximated by smooth curves using regression splines, coined functional data (see Section 2.4). Once the spectral curves have been approximated by means of regression splines, an FPCA has been applied according to Section 2.4.

**Figure 4:**
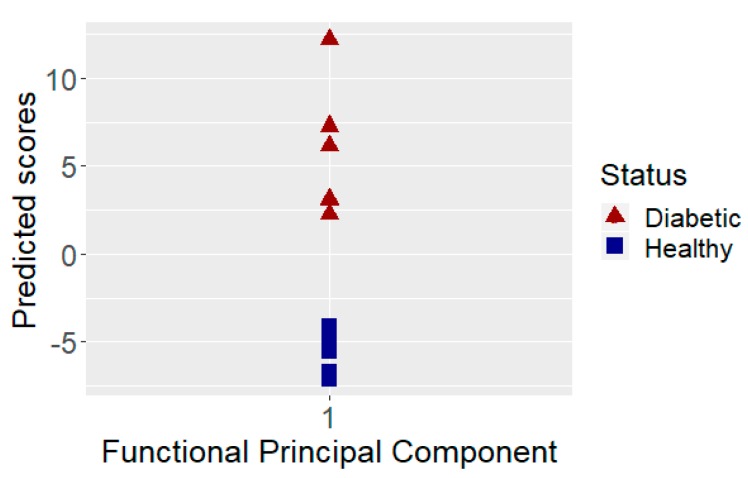
Predicted scores for the test sample, using only the first functional principal component.

Both the training sample and test sample were acquired using different signal power configurations due to different transmittance levels. As seen in Table 1, mice of the test sample were four months younger than the mice in the training sample. The presence of sustained medium/long-term hyperglycemia in animals led to dehydration problems and could reduce the thickness of the animal skin layers. The longer periods of hyperglycemia in animals led to higher transmittance levels than that of younger animals. Therefore, the transmission amplitude was normalized according to the mean of each sample. However, as shown in Figure 4, the correct discrimination of diabetic cases with sustained hyperglycemia was not affected. This supports the conclusion found in our previous publication [22] that the discrimination of sustained hyperglycemia was insensitive to the skin thickness of the measured mice, which directly relates the water content involved in the measure. It is important to note here that the training sample was measured during the year 2015 and, after calibration of the system, the test sample was blindly classified in March 2017. No changes in the set-up of the instrument were done after calibration and during the measurements of the test sample.

The blindly determined animal scores after proper calibration of the system using the training sample are shown in Figure 4. In this figure, we can see that taking into account only the first functional principal component estimated the training sample (which explains 99% of the total variance of the data) and 100% of the testing data set correctly assigned the animals. In Figure 4, we can see that the principal component assigns without errors positive scores to the diabetic mice and negative scores to the healthy mice. These results indicated excellent consistency and robustness of the sensing approach for diabetes discrimination for mice assessed during different experiments and with different age.

### 3.2. Temporal Evolution Study

**Figure 5:**
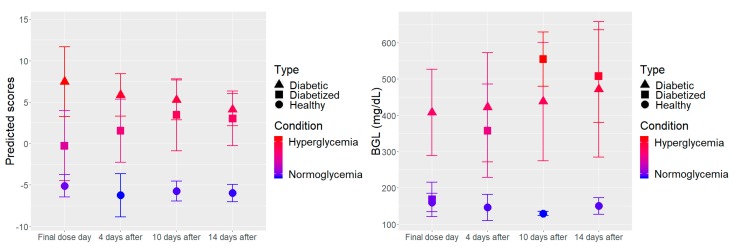
The mean score determined and the mean value of the measured blood glucose level for each group with the corresponding standard deviation are shown as a function of measurement days at the (**left panel**) and (**right panel**), respectively.

As mentioned in the introduction, the temporal evolution of glycemic states (specially sustained hyperglycemia) has not been reported yet using non-invasive techniques. With the described mm-wave spectroscopic sensing it was straightforward to measure the time evolution of the glycemic state of the various mouse groups, due to the stability of the instrument and the robustness of the FPCA approach, capable of blindly classifying the diabetic mice. Therefore, we have studied the sensitivity of the proposed approach to changes in glycemic states associated with diabetes on 16 mice with drug-induced diabetes (see Section 2.1). These mice were measured simultaneously with the healthy and diabetic mice of the previous subsection as control groups. The sample (16 diabetized mice, 6 healthy mice, and 6 diabetic mice) was evaluated at four different days along 14 days between the first and last measurement. Due to the fast metabolomic processes in mice, a duration of 14 days should give a clear indication of glycemic conditions in the animals. Measurements had started when the diabetes induction process was completed, hence, the first measurement corresponded to the final dose day (the fifth day after the application of the first dose, see Section 2.2). Then, using the approach validated before, the variability of the prompt detection of new cases of diabetes was evaluated. To this end, the mean value of the determined scores by groups, with the corresponding standard deviation for the test sample in the four measured days (final dose day, day 4, 10 and 14 later) are shown in Figure 5 (left panel). In this figure, we can clearly see that the diabetized mice evolved from scores between the diabetic and healthy mice towards the diabetic region of the positive scores. Such an evolution was confirmed by the evolution of the measured blood glucose levels for the diabetized mice at the same days, which are depicted in Figure 5 (right panel). Following both trajectories, it can be clearly deduced that the sensing approach can follow with great sensitivity and temporal accuracy the evolution from normoglycemia to hyperglycemia condition right from the beginning of the test.

It is important to remark that the biological response of each mouse to the diabetes induction process may vary as some mice are more resistant to the drug (Streptozotocin). This biological variation is also captured by the proposed sensing system, as depicted in Figure 5. Some diabetized mice were already identified as hyperglycemic at the final dose, even though the measured BGL is still very similar to the healthy group. This suggests that the sensing approach enhances the diagnostic capability to early detection of diabetes effects in sustained hyperglycemia. The remaining cases have a slower transition accounting for different speeds of development of the induced diabetes in mice. Nevertheless, 71% of the test sample were correctly assigned at the final dose day and four days later. After 10 and 14 days, the induced diabetes process has completed, 91% of the test sample were correctly assigned. The 80% of diabetized mice measured during the last day were detected as hyperglycemic. Moreover, it could be seen in Figure 5 (left panel) that the average predicted scores of diabetized and diabetic mice at the last two measurement days were very similar, especially at day 14. This indicates remarkable feasibility to identify diabetes in mice only two weeks after induction.

## 4. Discussion

In this work, a non-invasive and reliable method for the assessment of sustained hyperglycemia typically associated with diabetes by sensing the metabolism has been validated. The sensing method relies on a mm-wave transmission spectrometer and FPCA approach. The FPCA approach has been tested on a sample of 12 mice with a 100% correct assignment rate. Even though samples involved in the results had different transmittance levels and biological diversity (different mice strains), clear discrimination was achieved, irrespective of the individual skin properties, demonstrating the reliability of the approach for hyperglycemia detection. Furthermore, high sensitivity and temporal accuracy for the monitoring of glycemic changes in mice has been experimentally demonstrated by a longitudinal study on a group of sixteen mice with drug-induced diabetes. The stability of the mm-wave instrument together with the robustness of the FPCA approach allows us to clearly track the evolution of mice with drug-induced diabetes from normoglycemia to hyperglycemia, enabling the prompt detection of new hyperglycemia cases. These unprecedented results support the great potential of the proposed method not only for the diagnosis of diabetes but also for the early detection of diabetes and monitoring glycemia evolution in diabetics with a resolution of a few days. Although the minimum length of time required for the method to detect glycemic changes is still to be evaluated, the results indicate that different hyperglycemic states associated with diabetes are detected with only four days of uncontrolled occurrence. The obtained results indicate that the proposed sensing approach offers a comparable measure to that provided by the HbA1c test, but the lead times are very short compared to the periods required for the HbA1c test. Therefore, a correlation study is currently performed between the spectral measure presented here and the glycated hemoglobin value in patients suffering Type 1 Diabetes. The spectroscopic system provides results in seconds and it is a highly reliable instrument not requiring frequent calibration. Moreover, the system is not based on disposables or consumable materials, reducing substantially the operational costs, which can be made very compact and realized cost-efficiently. Therefore, we consider that the presented approach has great potential in developing a new non-invasive technique to improve medical proceedings for combating diabetes mellitus.

## Figures and Tables

**Table 1:** Mice strains employed for the validation test.

Sample	Mice strain	Condition	Variation	Expected Glucose Level (mg/dL)	Time with the Condition	Label	Quantity
Training sample	C57B16/J	Normoglycemia	-	100	6 months	Healthy mice	2
	BalbC	Normoglycemia	-	100	6 months	Healthy mice	2
	NMRI-Foxn1^nu^/Foxn1^nu^	Normoglycemia	-	100	1 month	Healthy mice	4
	Lep^ob^/Lep^ob^	Normoglycemia	Normoglycemic by leptin-pump	100	25 days	Healthy mice	2
	NMRI-Foxn1^nu^/Foxn1^nu^	Hyperglycemia	Drug-induced diabetes	>300	12 days	Diabetized mice	3
	Lep^ob^/Lep^ob^	Hyperglycemia	-	>150	6 months	Obese mice	5
	Lep^db^/Lep^db^	Hyperglycemia	-	>250	6 months	Diabetic mice	2
Test sample	C57B16/J	Normoglycemia	-	100	9 weeks	Healthy mice	6
	Lep^db^/Lep^db^	Hyperglycemia	-	>250	9 weeks	Diabetic mice	6

## Data Availability

The datasets generated and analyzed during the current study are available from the corresponding author on reasonable request.
